# The impact of the Orlando mass shooting on fear of victimization and gun-purchasing intentions: Not what one might expect

**DOI:** 10.1371/journal.pone.0182408

**Published:** 2017-08-11

**Authors:** Wolfgang Stroebe, N. Pontus Leander, Arie W. Kruglanski

**Affiliations:** 1 Department of Social and Organizational Psychology, University of Groningen, Groningen, Netherlands; 2 Department of Psychology, University of Maryland, College Park, Maryland, United States of America; Tampereen Yliopisto, FINLAND

## Abstract

Mass public shootings are typically followed by a spike in gun sales as well as calls for stricter gun control laws. What remains unclear is whether the spike in gun sales is motivated by increased threat perceptions or by concerns about gun control, or whether the sales are mainly driven by non-owners purchasing guns or gun owners adding to their collection. Two surveys of gun owners and non-owners, conducted immediately before and after the Orlando shooting, allowed us to assess its impact on threat perceptions and on gun-purchasing intentions. Although there was a minor impact on threat perceptions of non-owners, neither group reported any increased gun-purchasing intentions or an increased need of a gun for protection and self-defense. We suggest that these responses are representative for the majority of Americans and, therefore, people who are influenced by mass shootings to buy guns are probably an atypical minority.

## Introduction

There are two predictable and recurring reactions to any mass shooting: There is a call by politicians of the Democratic Party (and also of relatives of victims) for stricter gun control laws; this reflects a belief that tighter controls can help to prevent mass shootings [1]. Yet, at the same time, there is a spike in requests for FBI background checks; this suggests that others instead decide to buy guns, perhaps due to a belief that being well-armed can deter such crimes [2, 3]. It remains unclear whether these increased sales are motivated by increased threat perceptions or gun control concerns and whether they are due to non-owners purchasing guns or gun owners adding to their collection. To address these questions, we use data from two surveys of gun owners and non-owners, conducted immediately before and after the 2016 Orlando mass shooting.

## What is a mass shooting?

Before we address our research questions we must clarify our definition of a mass shooting because the association between a spike in gun sales and calls for stricter gun control was identified by journalists—who define mass shootings based on different criteria than researchers. We will thus review the different definitions of mass shootings that are used by journalists and researchers, because the choice of definition apparently varies by the purpose one pursues in studying mass shootings. Given that newspapers are mainly interested in the newsworthiness of a story, the number of people that are killed or wounded in an incident is probably the most important criterion. However, definitions of mass shootings may vary by the types of victims. If a researcher is interested in the motivation that drives people to commit such horrendous acts, then it makes little sense to lump together shooting incidents that have vastly different etiologies—targeted shootings such as familicides and felony-related shootings may involve different motivations than mass public shootings wherein an active shooter randomly kills strangers in some public space.

It is also unclear at what point the broader public recognizes an incident as a mass shooting. If you have ever wondered why different newspapers report wildly different numbers of mass shootings for a given year, the reason is that there is no generally accepted definition of mass shooting and that different databases disagree on nearly every aspect of such a definition [4]. The first point of disagreement is whether to count the number of people shot or the number of people killed. The Gun Violence Archive [5], a data base preferred by the New York Times and the Washington Post, uses the number of people *shot* rather than killed in an incident of violence as criterion for mass shooting. This definition is the most liberal. It is purely numerical and reflects all shootings which reach that statistical threshold of four individuals wounded or shot.

All other data bases define mass shootings in terms of the number of people *killed*, based on a definition of “mass murder” introduced by the FBI in 2005. The FBI defined “mass murder” as a multiple *homicide* in which *four* or more victims are murdered, within one event, and in one or more locations in close geographical proximity [6]. To complicate matters, in January 14, 2013 the U.S. Congress passed a note to the *Investigative Assistance for Violent Crimes Act of 2012*. 6 USC 101 that lowered the numerical criterion for mass murder to “*3 or more killings in a single incident*” [7]. And yet, even though the FBI adapted their definition of mass murder to mean three or more killing in a single incident” [8], the change in the FBI’s numerical criterion did not influence the definition of mass shootings used by Congress in a report of the Congressional Research Service about “Mass Murder with Firearms: Victims and Consequences 1999–2013” published in 2015 [9]. This report defined mass shootings as multiple homicides in “*which at least* ***four*** *victims are murdered with firearms*, *within one event*, *and in one or more locations in close proximity*”. The change in the numerical criterion did, however, influence another important source, namely the data base of mass shootings compiled by the news organization “Mother Jones”. From January 2013 onwards, Mother Jones lowered their inclusion criterion for mass shootings to three victims having been killed [10].

The Congressional Report also introduced an important distinction between mass shootings and mass *public* shootings, which became another point of disagreement [9]. The difference between mass shootings and mass public shootings is that mass public shootings exclude mass shootings where a family member kills at least four members of their family, and felony-related mass murders where the shooting of four people is related to some criminal activity. Mass public shootings are considerably less frequent than mass shootings, but claim more victims killed or wounded.

This exclusion of felony-related mass shootings and familicides can be justified in terms of motive and/or location [11]. Whereas an active shooter selects individual victims at random (sometimes from a category of people, e.g., Blacks, Jews, Muslims) in some public place, this is typically not the case in familicides or family-related killings. Familicides are usually the result of a domestic dispute and occur in private homes. Felony-related mass shootings may be gang-related or part of a robbery and are therefore also motivationally different from mass shootings. Whereas the Gun Violence Archive [5] does not exclude any type of gun violence such as gang shootings or domestic violence, the data base of Mother Jones excludes these types of mass killings [10]. However, in contrast to the Congressional Report [9], Mother Jones uses the lower numerical criterion from 2013 onwards.

These exclusion criteria also determine whether there is an increase in mass shootings over time. Whereas the frequency of mass shootings (i.e., including familicides and felony-related shootings) appears to have stayed relatively stable over time [12], there was a slow increase in mass *public* shootings between 1970 and 2013 [9]. According to the Congressional Report [6], there was one incident per year during the 1970s (with an average of 5.5 victims murdered and 2 wounded), but 4.5 incidents per year between 2010 and 2013 (7.4 victims killed and 6.3 victims injured per incident. Cohen, Azrael and Miller [11] even identified an acceleration in the rate of mass public shooting between the years 2011 to 2014. In contrast, Fox and DeLatour [12] demonstrated that there was no temporal increase in mass shootings (i.e., including familicides and felony-related shootings).

## Mass shootings and gun acquisition

Regardless of which definition one applies, the public mass shooting at the Pulse nightclub in Orlando, on June 12, 2016, in which 50 people died (including the gunman), and 53 were wounded, was the deadliest mass shooting by a single shooter in U.S. history [13]. Predictably, during that month, requests for background checks by the FBI’s National Instant Criminal Background Check System (NICS) reached an all-time monthly high [2]. Given that there is no federal registration system of gun sales, requests for NICS background checks are the best proxy. However, as background checks are also required for applications for a concealed carry license, the number of such requests might overestimate the number of actual gun sales. On the other hand, because private sales of guns—as well as many sales made at gun shows—do not require background checks, the monthly number of such background check requests could also underestimate the gun sales volume. And also, predictably, a week after the Orlando shooting—on June 18—President Obama had repeated his call for stricter gun control laws: *“Being tough on terrorism*, *particularly the sorts of homegrown terrorism that we've seen now in Orlando and San Bernardino*, *means making it harder for people who want to kill Americans to get their hands on assault weapons that are capable of killing dozens of innocents as quickly as possible”* [1].

As mentioned earlier, there are two potential reasons for the association of mass shootings with the increase in gun sales: It is possible that mass shootings increase people’s fear of crime and motivates them to buy a gun for self-protection; alternatively, the fact that politicians respond to mass shootings with a demand for stricter gun controls could increase people’s fear that the government will impose stricter gun control measures. These hypotheses will be discussed in the following sections.

## The fear of crime interpretation

The fear of crime interpretation consists of two assumptions: first, that people’s fear of crime motivates them to buy a gun for protection; and second, that mass shootings increase people’s fear of crime. The first assumption is consistent with the finding that 60 percent of gun owners cite protection and self-defense as main reason for owning a gun [3,14]. Yet surprisingly, the evidence for the fear of crime interpretation has not been all that consistent. In a review of that literature, Kleck et al., [15] note that *“Studies assessing the effect of fear/risk and criminal victimization on gun ownership have obtained wildly varying results” (p*. *313)*. They criticize previous research for methodological weaknesses, such as failure to find out whether a respondent is actually the owner of a gun (rather than being a household member) and whether the gun is actually a handgun (rather than some other type of gun). For example, William and McGrath [16], who measured gun ownership with the question whether respondents had any guns or revolvers in their home and fear of crime with the question whether respondents experienced fear when walking around their neighborhood at night, reported a negative association between the two variables. People who experienced greater fear were less likely to own a gun. One possible explanation is that gun ownership alleviates people’s fear. However, with the same measures of fear and gun ownership, McClain [17] found a weak positive association for white, but not black respondents. Hill, Howell and Driver [18], who operationalized protective gun ownership as “handgun owners, who do not hunt”, found that fear only related to protective gun ownership in men and not women. As Marciniak and Loftus [19] pointed out, their operationalization of protective gun ownership is problematic, because it assumes that hunting is inconsistent with owning a handgun for protection. It is thus difficult to draw any clear interpretations from this type of research with regards to how fear of crime correlates with gun ownership.

Findings of studies that directly measured personal gun ownership for protection also offer mixed support for the fear of crime hypothesis. For example, Cao et al. [20], who directly measured gun ownership for protection, found it positively associated with the (perceived) relative crime level in the person’s community, but unrelated to perceived risk of victimization or fear of victimization or fear of crime. One would have expected that fear of victimization or fear of crime would have mediated the need for self-protection. Again, it is possible that gun ownership made people feel less fearful. However, this explanation cannot account for the inconsistencies in findings reported by Lizotte and Bordua [21]: In their study, the only determinant of protective gun ownership was the respondents’ *objective* level of violent crime in their county; furthermore, although their respondents’ fear of crime was determined by experienced victimization and *perceived* high crime level in their community, they coped with this fear by installing home defense measures such as special locks or a burglar alarm rather than acquiring a gun.

Stronger support for the fear of crime hypothesis comes from studies by Kleck et al. [15] and Stroebe et al. [22]. Kleck et al reported a significant positive association between personal ownership of a handgun for protection and self-defense and perceived risk of crime. Similarly, in a recent survey of the motivational bases of American gun ownership, Stroebe et al. [22] found that gun ownership was predicted by the perceived likelihood of lifetime assault victimization (PLRA). Gun owners reported greater PLRA than non-owners [22]. PLRA was positively associated with the need for self-defense as a main reason for gun ownershp.

The Stroebe et al. study may also have uncovered one of the reasons for inconsistencies in previous studies of the association of protective gun ownership with fear of crime. They found that both gun ownership and owning a gun for self-defense were additionally influenced by a diffuse and *generalized* threat emanating from the belief that the world is a dangerous and unpredictable place, populated by mean people out to hurt others for no real reason (Belief in a Dangerous World—BDW) [23, 24]. And whereas PLRA was influenced by a respondent’s previous victimization experience, BDW was mainly determined by political orientation, with politically conservative respondents scoring higher.

Whereas empirical support for the first part of the fear of crime interpretation is mixed, the second part of the theory—that mass shootings increases people’s fear that they might become victim of such an incident—has never been tested. The evidence from opinion polls is indicative, but not conclusive. Although 48 percent of Americans reported a great deal or a fair amount of worry about future terrorist attacks according to a Gallup Poll conducted in March, 2015 [25], and although 41 percent said that they are at least somewhat worried that they or a family member will become a victim in a CNN/ORC poll conducted immediately after the Orlando shooting [26], we do not know the level of such worries immediately before the Orlando shooting. Furthermore, although it would seem plausible that the immediate affective response to a mass shooting would be to fear that this could happen to oneself or to a family member, the chance of becoming a victim of a mass shooting is exceedingly rare. For example, 31 victims were killed in the 5 mass shootings committed in 2013 according to the Congressional Report [6]. In contrast, during the same year, 11,208 gun homicides were committed [20].

### The fear of stricter gun control interpretation

Given that calls for stricter gun control laws have been a standard response by politicians to mass shootings, it is plausible that shootings *also* increase Americans’ fear that stricter gun control laws might be imposed. In 2016, the New York Times presented an extensive analysis that illustrated that gun sales increased after each call for stricter gun controls [27]. In fact, January 2013 –shortly after the re-election of President Obama—saw one of the highest gun sales on record, with two million guns sold. In contrast, whereas President Obama had been good for firearm sales, the election of Donald Trump has been bad for the gun industry. After record sales of guns in 2015, the election of Donald Trump resulted not only in a drop of gun sales, but also a substantial plunge in gun company stock values [28]. One day after the election, SturmRuger stocks fell 14% and Smith & Wesson (now American Outdoor Brands) fell by 15%. Thus, as Smith wrote in February 2017 in a piece for CNN Money “*President Barack Obama was the greatest gun salesman in America—until Hillary Clinton ran to replace him*. *Sales soared to records because gun owners feared they would impose tougher gun restrictions*. *Now that a Republican endorsed by the National Rifle Association is in the White House*, *those supposed villains have disappeared*. *Sales of guns and ammo are falling*, *right along with the stocks of gun makers*.*”* [29]. Members of minorities appear to be the only exception to this trend. The National African Gun Association has seen a recent surge in members which, according to its president Philip Smith, appears to be driven by fear that the nation’s divisive politics could spiral into violence. Between Election Day and the end of February, the group has added more than 7,000 members [28].

### Assessing the validity of the two interpretations

Given that both fear of crime and fear of stricter gun control appear to increase gun sales, it is unclear whether it is fear of crime *or* fear of stricter gun control laws (or both) that drives gun buying after a mass shooting. One—admittedly rather weak—way to evaluate the relative validity of these two hypotheses is to examine whether the gun buying intentions are immediate or delayed. Given that one would expect the effect of mass shootings on fear of crime to weaken over time, and given that calls for gun control would become stronger in the weeks following a mass shooting, a delayed spike is more likely to be due concerns about the imposition of stricter gun control laws. A recent study by Wallace [30] assessed the time interval between six mass shootings in the USA (from 2000–2010), and the spike in requests for background checks. For two of the mass shootings, she found that effects on requests for background checks spiked immediately (Northern Illinois University shooting, 02/14/2008; Kirkwood, Missouri, 7-02-2008); for the other four mass shootings, effect sizes spiked four to five months later (Wakefield, MA, 12/26/2000; Virginia Tech, 04/16/2007; Carthage, NC, 03/29/2009; Binghampton, NY, 04/03/2009.

A stronger test of the two interpretations would require information about the types of people who buy guns following a mass shooting. Because the spikes in requests for background checks [2, 22] are the only available indicators of the impact of mass shootings on gun sales, we do not know who buys guns—whether it is first-time gun buyers (i.e., non-owners), or current gun owners who want to add to their existing arsenal. According to a recent survey of U.S. gun owners, the average gun owner owns several guns (*M* = 4.06, *SD* = 4.37; *range*: 1–30) [22]. One could imagine the possession of four firearms would be sufficient to satisfy a need for self-defense. So maybe it is just those who had a preexisting intention to buy—such as hobbyists who already wanted to add a precision rifle or modern sporting rifle to their collection, who speed up their purchase for fear of stricter gun control laws. Thus, we would predict that if the spike in gun sales is caused by gun owners purchasing more guns, it is fear of stricter gun laws rather than fear of crime that drives their behavior. The fear of crime explanation appears to be more applicable to people who do not own a gun who decide―after a mass shooting—that it is time to buy one as a means of self-defense. The assumption is that a mass shooting increases fear of crime and as a result, the need for protection and self-defense.

## The Orlando mass shooting

It was by terrible coincidence that the deadliest mass public shooting in U.S. history occurred just as we were finishing a survey of U.S. gun owners and non-owners that had originally sought to assess the motives for gun ownership [22]. The grim circumstances offered a rare opportunity to study the impact a mass shooting has on gun-related belief systems of gun owners and non-owners, so we decided to repeat the survey to allow for a pre-post Orlando analysis using a between groups design. To avoid the possibility that respondents would remember their answer to the first survey, we collected data from a new, but comparable sample rather than re-contacting the same individuals. Our surveys included measures of the two threats assumed to motivate gun purchases (“Belief in a Dangerous World” and “Perceived Lifetime Risk of Assault”). We also assessed respondents’ need of a gun for self-defense and their perception of the effectiveness of a gun for self-defense. As a measure of people’s *expectations* regarding the introduction of stricter gun control laws, we assessed the belief the government wanted to take people’s guns away. Finally, we asked respondents for the amount of money they intended to invest in gun-related products over the next six months. Note that some of the variables we report in the present study also appear in our paper that addressed the original purpose of the survey: to develop and test a model of the motivational bases of gun ownership [22]. However, that study used those data merely to assess the stability of a two-component theory of gun ownership by replicating it in two samples. The study did not analyze the effects of the Orlando shooting on non owners nor did it report and analyze data on spending intentions. Thus, the two articles address different questions and review different literatures.

The history of this project also explains some of the limitations of this research in addressing the impact of the Orlando mass shooting on fear of crime and gun spending intentions. Given that the project was conducted to develop and test a psychological model of the motivational bases of gun ownership, we focused only on men because they still represent the majority of gun owners [31]. Furthermore, given that we wanted to use the post-Orlando sample to assess the stability of our models after such a horrendous tragedy, we opted for a new sample rather than attempting to re-contact the original, pre-Orlando survey respondents. However, we made sure that both samples are generally comparable with regard to relevant demographic criteria. Regrettably, we did not stratify our sample by race.

## Method

### Ethical approval

The study was approved by the Ethical Committee Psychology (ECP) of the Faculty of Social Sciences of the University of Groningen, The Netherlands. Participants were given the following consent statement: “*This university-based psychological study will ask about your beliefs*, *attitudes*, *and experiences regarding gun ownership and the use of firearms*. *Your participation is completely anonymous*. *No identifying information will be collected from you*. *Only members of the research team will have access to the survey data*, *but even they cannot link the data to any single person*. *You can decide whether or not to participate in the study*. *You can leave the study at any time*.*”*

### Participants

One thousand seven hundred thirty men, in the United States, were recruited via the market research firm Qualtrics Panels, to complete the study online between May 31 and June 22, 2016. No data were collected on June 12 –the day of the mass shooting. Of these participants, n = 787 completed the study before the mass shooting and n = 943 completed the study after. An additional n = 37 were excluded to do quality problems, n = 108 declined to answer the gun spending intention item, and two gave unlikely values on the gun spending intention item (> 8 *SD* beyond the mean).

Participants were recruited primarily by gun ownership (n = 847 gun owners, n = 883 non-owners), with maximum quotas for region of country, age, education, and income. Regions included: Midwest (n = 402), West (n = 330), Northeast (n = 326) and South (n = 672). Median age category was *“35–44*,*”* education was “*Some college*,” and income was $35,000-$50,000 per year. The distribution of education did not differ between gun owners and non-owners (*F* < 1), but gun owners tended to report slightly higher age and income categories (*F*_*age*_ = 10.86, *p* = .001, *η*^2^_p_ = .006; *F*_*income*_ = 37.29, *p* < .001, *η*^2^_p_ = .021). Among the gun owners, 79.8% (n = 676) owned a handgun and 75.8% (n = 642) owned a long gun. The mean number of guns owned was 3.71 (*SD* = 4.06; range: 1–30), which reflects a pre-post Orlando difference in self-reported gun ownership. Twenty-one declined to report number of guns owned, but this did not reliably differ by pre-post Orlando (*t* < 1). To assess whether there were significant differences between our pre- and post-Orlando samples of gun owners and non-owners, we conducted pre-post Orlando comparisons. Gun owners and non-owners did not differ significantly in age, income or education (*F*s < 1). However, for gun owners, there was a marginally lower likelihood of being from the South for members of the post-Orlando sample (Wald = 2.86, *p* > .09); note, however, that the result is only marginal and we placed quotas on the number of Southerners to recruit. Non-owners in the post Orlando sample were also slightly older (*F* = 6.37, *p* = .012, *η*^2^_p_ = .007). Ultimately, these relatively minor differences in region and age had no bearing on any of the results reported below when included as covariates.

### Procedure

Participants first self-reported their demographics, above, to screen them for gender (males only), gun ownership *(“Do you own a gun*?”), and to ensure a wide range of demographics (based on region, age, education, and income). To minimize biased language or terminology, the questionnaires were designed with feedback from two professionals in gun sales and manufacturing.

Participants then reported their threat-related beliefs and gun ownership, in counterbalanced order. In the post-Orlando sample, knowledge of the mass shooting was an inclusion criterion.

Perceived lifetime risk of assault. Participants were asked, *“What do you estimate is the likelihood the following will happen in your lifetime (in your future)*?*”* There were three items: *“You will be mugged”*; “*You will be violently attacked*, and *“Your home will be invaded by an armed burglar”* (rated *1* = *Not likely at all*, to *7* = *Very likely*, α = .89). The post-Orlando version of the study included a fourth item, *“You will be present during a mass shooting”* (*M* = 2.61, *SD* = 1.58).

Belief in a dangerous world. To assess participants’ belief in a dangerous world (BDW), we used the 10-item version of Altemeyer’s [23] BDW scale developed by Duckitt [24]. Participants indicated their agreement with statements such as *“There are many dangerous people in our society who will attack someone out of pure meanness*, *for no reason at all”* or *“Any day now*, *chaos and lawlessness could erupt around us*. *All signs are pointing to it*.*”* (rated 1 = *Strongly disagree* to 7 = *Strongly agree*, α = .84). The gun industry professionals advising us on the study recommended we avoid use of the term “anarchy,” in item 2 of the BDW scale, so we replaced it with “lawlessness”. Note that the BDW was included in the analysis because Stroebe et al. [22] demonstrated that, in addition to the specific threat of being violently attacked, gun ownership is also motivated by the generalized threat emanating from the belief that the world is a dangerous and unpredictable place.

Main reasons for owning a gun. Gun owners reported the extent to which each of the following was a reason they owned a gun: *Protection/Self-defense*, *Hunting*, *Target/Sport Shooting*, *Constitutional right / 2*^*nd*^ *Amendment*, *Collecting guns / Hobby*, and *Other* (rated *1* = *Not important / Not applicable* to *7* = *Very important*). The questions were reframed for non-owners to be about the main reasons they might consider buying a gun.

Belief gun possession could have prevented the mass shooting. In the post-Orlando version of the study, participants read, *“The mass shooting in Orlando might have been prevented if…”* and below were several different possibilities (rated *-3* = *Very doubtful/Not applicabl*e to +*3* = *Very possible*). The prevention belief of interest was *“…people at the nightclub were armed*.*”* The other items were *“stricter gun control laws were in place”*, *“better mental health care existed”*, *“society was more accepting of gays”*, *“there was more surveillance of suspected radicals*,*”* and *“society was more cautious of Muslims*.*”*

Unrelated task. Participants then completed a cognitive task lasting about five minutes, wherein we manipulated its difficulty and subsequently measured state anxious, hostile, and quiescent affect. This manipulation had the expected main effects on the affect measures (*F*s ≥ 49.75, *p*s < .001), but no main effects on any the variables tested here (*F*s < 3.2, *p*s ≥ .077).

Perceived effectiveness of firearms. All participants were asked, *“How effective is gun possession as a means of…”* and were first presented with our motivation of interest, *“Protection and Self-Defense”* (rated 1 = *Not effective at all* to 7 = *Extremely effective*).

Attitudes towards guns. Participants then rated their attitude towards each of four categories of firearms: Handguns, Precision Rifles, Modern sporting Rifles, and Shotguns (*rated -3* = *Extremely negative* to *+3* = *Extremely positive*, α = .91).

Sociopolitical beliefs. Among a series of other questions about their beliefs and experiences with gun ownership and use, participants were asked *“In general*, *do you believe the laws covering the sale of firearms should be made more strict*, *less strict*, *or kept as they are now*?*”* (rated *1* = *Much less strict* to *7* = *Much more strict*). They also rated their agreement with “*In general*, *if more people had guns*, *there would be less crime”* and *“In general*, *the government wants to take people’s guns away”* (rated *1* = *Strongly disagree* to *7* = *Strongly agree*). “

Experience with victimization. Participants completed a series of questions related to their personal experience with guns and crime, including whether they knew a specific instance in which someone close to them was the victim of violent crime (coded 1 if they selected *“family member”*, *“close friend”*, *“neighbor”*, *“someone else close* [text-entry option]*”*, or coded 0 for *“No”* or *“Not sure”*). Each participant also answered whether he had ever been a victim of a violent crime himself (coded 1 for *Yes*, 0 for *No*).

Investment intentions. All participants were asked how much money they intended to invest in gun-related products (including purchase of a gun) during the next 6 months. These values were log-transformed to adjust for positive skew.

## Results

Table 1 presents the mean responses of gun owners and non-owners surveyed immediately before and after the Orlando mass shooting. The table also reports the results of significance tests for differences between pre-Orlando and post-Orlando responses. We conducted separate one-way ANOVAs within the gun owner and non-owner groups on each of the relevant measures. The Orlando mass shooting had only a minor impact on non-owners’ perceptions of threat: Although non-owners believed the world to be marginally more dangerous after Orlando (BDW, *M*_*post-Orlando*_ = 4.29 vs. *M*_*pre-Orlando*_ = 4.14, *F*(1, 881) = 3.66, *p* = .056, *η*^2^_p_ = .004), the mass shooting did not increase their perceived lifetime risk of being violently attacked (PLRA, *M*_*post-Orlando*_ = 3.29 vs. *M*_*pre-Orlando*_ = 3.18, *F*(1, 881) = 1.04, *p* = .308, *η*^2^_p_ = .001). Furthermore, the marginal increase in BDW did not correspond with any increased need to own a gun for self-defense, *M*_*post-Orlando*_ = 5.25 vs. *M*_*pre-Orlando*_ = 5.23, *F*(1, 881) = 0.02, *p* = .894, *η*^2^_p_ = .000. In fact, post-Orlando, non-owners perceived guns as *less* effective means for self-defense *M*_*post-Orlando*_ = 5.03 vs. *M*_*pre-Orlando*_ = 5.40, *F*(1, 881) = 9.28, *p* = .002, *η*^2^_p_ = .010; accordingly, their attitude towards guns, which was already not very positive, showed a negative shift post-Orlando *M*_*post-Orlando*_ = -0.06 vs. *M*_*pre-Orlando*_ = 0.48, *F*(1, 881) = 22.22, *p* < .001, *η*^2^_p_ = .025. After Orlando, non-owners were in favor of greater restrictions on gun sales, *M*_*post-Orlando*_ = 5.43 vs. *M*_*pre-Orlando*_ = 4.93, *F*(1, 881) = 22.22, *p* < .001, *η*^2^_p_ = .025 but, unexpectedly, less supportive of gun free zones, *M*_*post-Orlando*_ = 4.65 vs. *M*_*pre-Orlando*_ = 4.99, *F*(1, 881) = 5.81, *p* = .016, *η*^2^_p_ = .007). There was no shift in support for a federal gun registry (*F* < 1.4, *p* > .239).

**Table 1:** Means by gun ownership and Orlando. Asterisks represent a significant pre-post-Orlando difference (within gun ownership group). ** p < .01, * p < .05, ^†^ p < .06.

Variable	Non-owners *M (S*.*E*.*) Pre-Orlando*	*Post-Orlando*	Gun owners *M (S*.*E*.*) Pre-Orlando*	*Post-Orlando*
**Ever considered owning a gun**	4.33 (.11)	3.90 (.10)**	-	-
**Belief in Dangerous World** (10-item)	4.14 (.05)	4.29 (.06)^†^	4.43 (.06)	4.33 (.05)
**PLRA** (3-item)	3.18 (.07)	3.29 (.07)	3.78 (.08)	3.53 (.07)*
**Reason: Self-defense**	5.23 (.10)	5.25 (.09)	5.94 (.10)	5.92 (.09)
**Effectiveness of guns for:** *protection and self-defense*	5.40 (.09)	5.03 (.08)**	6.13 (.08)	6.08 (.07)
**Attitude towards guns** (4-item)	0.48 (.08)	-.06 (.08)**	1.85 (.06)	1.66 (.06)*
**Support for stricter laws on gun sales**	4.93 (.08)	5.43 (.07)**	4.66 (.08)	4.60 (.07)
**Support for gun-free zones**	4.99 (.10)	4.65 (.10)*	4.83 (.11)	4.75(.10)
**Belief: more guns would reduce crime**	3.91 (.10)	4.03 (.10)	3.84 (.11)	3.85 (.10)
**Belief: government wants to take guns away**	4.14 (.11)	4.26 (.10)	4.19 (.11)	4.40 (.10)
**Political orientation** (→liberal)	4.76(.11)	5.09 (.10)*	4.60 (.12)	4.57 (.10)
**Gun spending intentions (log$)**	1.83 (.13)	1.14 (.12)**	4.76 (.13)	4.41 (.12)*
**Intent to spend *any* money on guns**	31.4% (2.1)	20.2% (1.9)**	82.4% (2.0)	79.9% (1.8)

Finally, there is a significant decrease in non-owners’ intentions to spend money on gun-related products during the next six months, *M*_*post-Orlando ($log)*_ = 1.14 vs. *M*_*pre-Orlando ($log)*_ = 1.83, *F*(1, 881) = 15.91, *p* < .001, *η*^2^_p_ = .018. This latter effect becomes even clearer when examining the percentage of non-owners who intend to spend *any* money on guns: Whereas 31% of non-owners reported some level of spending intentions before Orlando, spending intentions dropped to 20% in the post Orlando sample. Now, part of the results could be due to other differences between the two samples: Post-Orlando non-owners also reported lower scores on whether they had ever *considered* owning a gun, which could suggest a sample difference, *M*_*post-Orlando*_ = 3.90 vs. *M*_*pre-Orlando*_ = 4.33, *F*(1, 881) = 8.04, *p* = .005, *η*^2^_p_ = .005. Yet if we control for this item, all the effects reported above remained significant—with the exception of political orientation, which was rendered marginally significant, *M*_*post-Orlando*_ = 5.09 vs. *M*_*pre-Orlando*_ = 4.76, *F*(1, 881) = 2.85, *p* = .092, *η*^2^_p_ = .003. To summarize, there appeared to be a negative effect of the Orlando mass shooting on non-owners’ gun spending intentions.

The Orlando mass shooting seemed to have had little impact on gun owners. There is no evidence of an increase in their fear of crime; Orlando did not increase BDW or PLRA—that is, belief in a dangerous world, *F*(1, 845) = 2.11, *p* = .147, *η*^2^_p_ = .002, or perceived lifetime risk of assault, *M*_*post-Orlando*_ = 3.53 vs. *M*_*pre-Orlando*_ = 3.78, *F*(1, 845) = 5.10, *p* = .025, *η*^2^_p_ = .006. In fact, it appeared to have decreased their perceived lifetime risk of assault—though this is probably because we added a fourth item about the specific risk of a mass shooting. Gun owners’ already-low support for stricter gun laws remained unaffected by the Orlando massacre, *F*(1, 845) = 0.28, *p* = .597, *η*^2^_p_ = .000. They also showed no difference in support for gun free zones, *M*_*post-Orlando*_ = 4.75 vs. *M*_*pre-Orlando*_ = 4.83, *F*(1, 845) = 0.24, *p* = .624, *h*^2^_p_ = .000. The Orlando mass shooting also failed to change their belief that more guns would lower crime, *F*(1, 845) = .001, *p* = .980, *η*^2^_p_ = .000. Notably, post-Orlando, the belief that guns reduce crime correlated positively with a belief the mass shooting could have been prevented if guests at the nightclub had been armed (*r* = .61, *p* < .001). Finally, the Orlando mass shooting failed to increase their perceived likelihood that the government would take their guns away, *F*(1, 845) = 1.84, *p* = .176, *η*^2^_p_ = .002.

The only surprising effects were that post-Orlando gun owners reported less positive attitudes, *M*_*post-Orlando*_ = 1.66 vs. *M*_*pre-Orlando*_ = 1.84, *F*(1, 845) = 5.03, *p* = .025, *η*^2^_p_ = .006, and even slightly lower intentions to invest money in gun-related products, *M*_*post-Orlando ($log)*_ = 4.41 vs. *M*_*pre-Orlando ($log)*_ = 4.76, *F*(1, 845) = 3.91, *p* = .048, *η*^2^_p_ = .005; however, these too could be partly due to sampling: post-Orlando gun owners also reported *owning* fewer guns, *F*(1, 824) = 6.73, *p* = .010, *η*_*p*_^*2*^ = .008, and controlling for number of guns owned rendered non-significant the pre-post Orlando effect on attitudes, *F*(1, 823) = 2.94, *p* = .087, *η*^2^_p_ = .004, and spending intentions, *F*(1, 823) = 2.06, *p* = .152, *η*^2^_p_ = .002 (note that controlling for this variable had no bearing on the other results).

Given that the Orlando mass shooting occurred in a gay nightclub, we also explored the possibility that it had greater impact on the fear of being in a mass shooting of gay rather than heterosexual men. Toward the end of the post-Orlando survey, participants had the option to indicate whether or not they were gay. Approximately 5.4% of those who responded to this question (i.e., 47 out of 867) reported being gay (of which 20 were gun owners and 27 were non-owners). A 2 (gun owner vs. non-owner) x 2 (gay vs. not gay) ANOVA on perceived risk of being in a mass shooting indicated no main effect of being gay, (*M*_*gay*_ = 2.83, *SD* = 1.67, *M*_*not gay*_ = 2.60, *SD* = 1.58), *F*(1, 864) = 1.16, *p* = .281, *h*^2^_p_ = .001, and no interaction with gun ownership (*F* < 1). Thus, after Orlando there was no indication that gay men were more likely than heterosexual men to perceive themselves to be at risk of being in a mass shooting.

Did past experience with crime victimization moderate reactions to Orlando? Victimization experiences was assessed by asking respondents whether they had ever been the victim of a violent crime or known somebody who had been victimized. With regards to participants’ own past victimization, we conducted 2 (Orlando) x 2 (Past Crime Victimization) analyses of variance (ANOVAs) separately for the gun owner and non-owner samples on spending intentions. Among the gun owners, there was neither a main effect of pre-post Orlando, *F*(1, 843) = 0.49, *p* = .482, *η*^2^_p_ = .001, nor a two-way interaction of Orlando and past crime victimization, *F*(1, 843) = 1.27, *p* = .260, *η*^2^_p_ = .002. There was instead just a marginal main effect of past crime victimization, *M*_*victims ($log)*_ = 4.94 vs. *M*_*non-victims ($log)*_ = 4.30, *F*(1, 843) = 3.83, *p* = .051, *η*^2^_p_ = .005. The more gun owners had experienced crime victimization in the past, the more they intended spending on guns, irrespective of the Orlando mass shooting. Among non-owners, there was the same negative main effect of Orlando on gun spending intentions reported in an earlier analysis (which was not observed among gun owners), *F*(1, 879) = 6.00, *p* = .015, *η*^2^_p_ = .007, the same positive main effect of past crime victimization as observed for gun owners, *M*_*victims ($log)*_ = 2.03 vs. *M*_*non-victims ($log)*_ = 1.38, *F*(1, 879) = 6.06, *p* = .014, *η*^2^_p_ = .007, but no interaction of Orlando and past crime victimization, *F*(1, 879) = 0.07, *p* = .786, *η*^2^_p_ = .000. Altogether, past crime victimization predicted higher spending intentions generally, irrespective of the Orlando mass shooting. Nearly identical results were observed when replacing participants’ own victimization experience with whether or not they just *knew* someone who had been a victim of crime (e.g., neighbor, close friend, family member). Altogether, past victimization did not moderate the impact of Orlando on participants’ gun spending intentions.

Given that the lack of an increase in spending intentions is inconsistent with the idea of a spike in gun sales after mass shootings, we considered whether at least hobbyists and gun collectors increased their spending intentions out of fear that the government will impose stricter gun control measures. We regressed spending intentions on gun ownership (1 = gun owner, -1 = non-owner), pre- vs. post-Orlando (1 = post-Orlando, -1 = pre-Orlando), *hobby/collecting guns* as a main reason for gun ownership (standardized), and all interactions. Results indicated only direct effects of gun ownership, B = 1.29 (95% CI: 1.17; 1.42), *t*(1713) = 20.37, *p* < .001, Orlando, B = -.25 (95% CI: -.37, -.12), *t*(1713) = -3.91, *p* < .001, and *hobby/collecting guns*, B = .79 (95% CI: .66; .92), *t*(1713) = 12.08, *p* < .001; there were no interactions (-1.7 < *t*s < 1.2, *p*s > .10). Thus, even though gun collectors have higher spending intentions than non-collectors, Orlando did not increase hobbyists’ spending intentions.

In a final analysis, we explored whether different types of gun ownership predicted a shift in spending intentions pre-post Orlando. From a fear of crime perspective, handgun ownership may be the best means of self-defense given their compact size and portability into public spaces; thus, gun owners who already own a handgun may not perceive as strong a need to buy another gun than owners of long guns only (e.g., those who own a shotgun or rifle) [32]. After Orlando, gun owners who were also handgun owners (n = 676) only showed a marginally reduced intention to spend any money on guns, *M*_*post-Orlando ($log)*_ = 4.61 vs. *M*_*pre-Orlando ($log)*_ = 4.98, *F*(1, 674) = 3.65, *p* = .057, *η*^2^_p_ = .005; owners of long guns only (n = 171) showed no difference in gun spending intentions, *M*_*post-Orlando ($log)*_ = 3.69 vs. *M*_*pre-Orlando ($log)*_ = 3.76, *F*(1, 169) = 0.03, *p* = .857, *η*^2^_p_ = .000. There was thus no indication that the Orlando shooting increased gun spending intentions among owners of specific types of weapons.

## Discussion

Even though the Orlando mass shooting was the deadliest mass shooting in American history, it failed to have an impact on respondents’ perceptions of their life-time risk of falling victim to violence, even if they had past experience with victimization. Given that gun owners already owned multiple guns and were thus unlikely to need another firearm for self-defense, we had expected that the spike in background checks typically observed after mass shootings (including Orlando) would be driven by non-owners deciding to buy a gun for the first time. Although the mass shooting slightly increased non-owners’ belief that the world is a dangerous place, the increased BDW did not correspond with an increased need to own a gun for self-defense, probably because the mass shooting decreased their perception of the effectiveness of gun possession for self-defense. After all, guns are of little use in mass shootings: experts warn against drawing a gun in an active shooter incident, because the police—or other concealed-carry gun owners—might mistake a would-be hero for the active shooter and kill him or her [33]. Non-owners also adopted more negative attitudes towards guns after Orlando. Ultimately, there was no evidence that the mass shooting motivated them to purchase a firearm. In fact, after Orlando, fewer non-owners reported even considering spending money on purchasing a gun.

There was also no evidence that the Orlando shooting motivated gun owners to increase their gun-related spending above and beyond spending they might have already planned before the shooting. This was even true for hobbyist gun owners and owners of long guns only (i.e., those who did not yet have a handgun). Given that the Orlando mass shooting did not increase gun owners’ threat perceptions, or their need to own a gun for protection and self-defense, the finding that the mass shooting did not increase their spending intentions is consistent with our theory that perceived threats precede defensive gun ownership [22]. More importantly, however, these findings are inconsistent with a fear of crime interpretation of increased gun sales after a mass shooting. Given that we also failed to find an impact of the Orlando mass shooting in respondents’ support for stricter gun control, there is no support for the assumption that the spike in background checks following the Orlando shooting was motivated by gun owners’ fear that the government would introduce stricter gun control legislation.

So how can we account for the inconsistency of our findings with reports of increased requests for background checks after the Orlando mass shooting? One possibility is that our results are not an effect of the Orlando shooting, but an artifact that reflects preexisting differences between the pre- and post-Orlando samples. The post-Orlando gun owners reported having fewer guns than the owners in the pre-Orlando sample. If people have fewer guns, they are likely to have lower spending intentions on gun-related products. However, even though controlling for this difference affected reported spending intentions, it only eliminated the apparent *reduction* in spending intentions. There was still no evidence of any increase in spending intentions.

There were also differences between the pre- and post-Orlando samples of non-owners. Non-owners in the post-Orlando sample rated their political orientation as somewhat more liberal (*M* = 5.08, *SE* = 0.09) than did the pre-Orlando sample (*M* = 4.71, *SE* = 0.11). Consistent with this, they reported also to have been less likely to have ever considered buying a gun. However, controlling for these pre-post Orlando differences did not extinguish the negative effect of Orlando on non-owners’ spending intentions.

Another possibility to reconcile our findings with the increase in requests for background checks following a mass shooting, is that these background checks were requested for approval of a concealed carry permit rather than the purchase of a gun. There are now 12.8 million concealed carry permits in the U.S.A. and in 2014 alone, 1.7 million new permits were issued [34]. It is possible that gun owners (against the advice of experts) believe a concealed weapon would offer them protection in a mass shooting. News of a mass shooting might, therefore, have motivated gun owners to apply for a concealed weapon permit. This could certainly account for part of the increase in requests for background checks. Yet it seems doubtful that the total increase in these requests was due to gun owners applying for concealed weapon permits; even if it were, it would imply that mass shootings do not increase gun-purchasing intentions.

We think that the most plausible explanation of our findings is that they represent the response of a vast majority of Americans to the Orlando mass shooting, and that the people responsible for the increase in background checks are of an atypical minority, too small to have a significant impact on our findings. According to press reports, which are based on a comparison of the number background checks requested in June 2016 (i.e. immediately after the Orlando mass shooting) with those in the same month of the previous year, there was an increase of 600,000 requests [2]. However, this increase might not be fully attributable to the Orlando shooting, because the anticipation of a Clinton presidency may have also helped to facilitate a general increase in background checks in 2016 (27,538,657), as compared to 2015 (23,141,970) [35]. Thus, even without Orlando there would have been more requests for background checks in June 2016 than in June 2015. But even if one assumes that 600,000 Americans decided to buy guns following the Orlando shooting, it is unlikely that sampling from approximately 245 million *adult* Americans, we would have captured a sufficient proportion of those 600,000 individuals to find a significant difference in gun spending intentions. This suggests that for most Americans, this tragic event had no impact either on their fear of crime or their fear of stricter gun control measures. This would be rational, because, as mentioned earlier, the chance of falling victim to a mass shooting is minimal. Similarly, as past experience has shown, there is virtually no chance that mass shootings would persuade a Republican-controlled Congress to introduce stricter gun control measures (even though matters might be different at a state level).

Given that we did not anticipate that there would be a mass shooting just as we had finished collecting data for a study on the motivational bases of gun ownership [22], our study was not optimally designed for the questions addressed in this article. As a result, our conclusions rest on less than firm empirical grounds. To generalize our findings to the US population would have required a representative sample and a repeated measures design. However, with our large pre-Orlando sample from all parts of the USA and stratified in terms of level of education and age as well as the extent of matching between the pre-post-Orlando samples, the effects of the Orlando mass shooting on fear of crime and gun control beliefs would have had to be extremely weak and/or very limited to not have registered in our survey. Furthermore, given that the chance is small that there will be another mass shooting just after a representative survey of gun ownership motives has been conducted, our data will probably be the most valid indication of the (non-)impact of a mass shooting on fear of crime or fear of restrictive gun control measures that will be available for the near future.

To optimally assess the motives of individuals who purchase guns following a mass shooting, one would need population studies just before and just after a mass shooting. Methodologically less stringent—but easier to achieve—would be interviews with people purchasing guns in the aftermath of a mass shooting. These respondents could be asked whether they had decided on their purchase because of the mass shooting and whether it was fear of crime and/or fear of stricter gun control measures that influenced their decision.

Despite some shortcomings in design, the findings of our study suggest that the widely held assumption that Americans respond to mass shootings with greater fear of both crime of stricter gun controls remains as unsupported as the assumption that large numbers of Americans buy guns following a mass shooting. There is no evidence of such a response in the large sample of Americans who answered our survey. It appears that the individuals who buy guns following a shooting are some kind of minority who are not representative of the American people. These unexpected findings should go a long way to reducing the bafflement expressed by people who wonder how mass shootings could motivate people to buy more guns. As Hudson [36] wrote in *The Atlantic* after the Aurora mass shooting, “*The Rest of the First World Is Astounded by America's Enduring Gun Culture*.*”* From their perspective, there is no better demonstration of the negative effects of the widespread availability of guns than a mass shooting.
